# Supplementary material for: Effects of growing Coptis chinensis Franch in the natural understory vs. under a manmade scaffold on its growth, alkaloid contents, and rhizosphere soil microenvironment
Source: PeerJ. 2022 Jul 20;10:e13676. doi: 10.7717/peerj.13676 (PMC9308463; doi:10.7717/peerj.13676)
Supplement: Supplemental Information 4 [file peerj-10-13676-s004.docx]

Table S4 Redundancy analysis on soil physicochemical properties and dominant fungal phyla

| Factor | RDA1 | RDA2 | r^2^ | Pr(>r) |
| --- | --- | --- | --- | --- |
| Organic matte | -0.987 | 0.164 | 0.074 | 0.370 |
| pH | 0.700 | -0.715 | 0.507 | 0.000 |
| Soil bulk | 0.735 | -0.679 | 0.409 | 0.001 |
| Total nitrogen | -0.231 | 0.973 | 0.114 | 0.187 |
| Total phosphorus | -0.526 | 0.850 | 0.053 | 0.478 |
| Total potassium | -0.569 | 0.822 | 0.034 | 0.588 |
| Available nitrogen | -0.645 | 0.764 | 0.246 | 0.016 |
| Available Phosphorus | 0.168 | 0.986 | 0.015 | 0.864 |
| Available potassium | -0.543 | -0.840 | 0.444 | 0.004 |
