# Supplementary material for: Effects of growing Coptis chinensis Franch in the natural understory vs. under a manmade scaffold on its growth, alkaloid contents, and rhizosphere soil microenvironment
Source: PeerJ. 2022 Jul 20;10:e13676. doi: 10.7717/peerj.13676 (PMC9308463; doi:10.7717/peerj.13676)
Supplement: Supplemental Information 5 [file peerj-10-13676-s005.docx]

Table S5 Redundancy analysis on soil physicochemical properties and dominant bacterial phyla

| factor | RDA1 | RDA2 | r^2^ | Pr(>r) |
| --- | --- | --- | --- | --- |
| Organic matte | 0.426 | 0.905 | 0.298 | 0.005 |
| pH | -0.137 | -0.991 | 0.694 | 0.000 |
| Soil bulk | 0.981 | -0.195 | 0.380 | 0.001 |
| Total nitrogen | 0.554 | 0.833 | 0.353 | 0.002 |
| Total phosphorus | 0.530 | 0.848 | 0.561 | 0.000 |
| Total potassium | -0.480 | 0.877 | 0.030 | 0.638 |
| Available nitrogen | -0.017 | 1.000 | 0.320 | 0.004 |
| Available Phosphorus | 0.741 | 0.671 | 0.603 | 0.000 |
| Available potassium | 0.739 | 0.673 | 0.390 | 0.003 |
