# Supplementary material for: Effects of growing Coptis chinensis Franch in the natural understory vs. under a manmade scaffold on its growth, alkaloid contents, and rhizosphere soil microenvironment
Source: PeerJ. 2022 Jul 20;10:e13676. doi: 10.7717/peerj.13676 (PMC9308463; doi:10.7717/peerj.13676)
Supplement: Supplemental Information 6 [file peerj-10-13676-s006.docx]

Table S6 Redundancy analysis on enzyme activity and soil dominant fungal phylum

|  | RDA1 | RDA2 | r^2^ | Pr (>r) |
| --- | --- | --- | --- | --- |
| Polyphenol oxidase | -0.737 | -0.676 | 0.153 | 0.121 |
| Dehydrogenase | 0.982 | 0.187 | 0.123 | 0.174 |
| Catalase | -0.994 | 0.106 | 0.038 | 0.591 |
| Peroxidase | -0.892 | 0.452 | 0.349 | 0.005 |
| Neutral protease | -0.686 | -0.727 | 0.387 | 0.001 |
| invertase | 0.959 | 0.285 | 0.473 | 0.000 |
| Neutral phosphatase | 0.929 | -0.371 | 0.051 | 0.499 |
| Urease | -0.991 | 0.133 | 0.183 | 0.076 |
