# Supplementary material for: Effects of growing Coptis chinensis Franch in the natural understory vs. under a manmade scaffold on its growth, alkaloid contents, and rhizosphere soil microenvironment
Source: PeerJ. 2022 Jul 20;10:e13676. doi: 10.7717/peerj.13676 (PMC9308463; doi:10.7717/peerj.13676)
Supplement: Supplemental Information 7 [file peerj-10-13676-s007.docx]

Table S7 Redundancy analysis on enzyme activity and soil dominant bacterial phylum

|  | RDA1 | RDA2 | r^2^ | Pr(>r) |
| --- | --- | --- | --- | --- |
| Polyphenol oxidase | -0.966 | -0.257 | 0.060 | 0.445 |
| Dehydrogenase | 0.986 | -0.164 | 0.344 | 0.004 |
| Catalase | 0.469 | 0.883 | 0.189 | 0.073 |
| Peroxidase | -0.983 | 0.183 | 0.180 | 0.073 |
| Neutral protease | -0.724 | 0.689 | 0.078 | 0.339 |
| invertase | 0.977 | -0.211 | 0.518 | 0.000 |
| Neutral phosphatase | -0.052 | -0.999 | 0.171 | 0.081 |
| Urease | -0.969 | 0.245 | 0.058 | 0.430 |
